# Therapeutic Potential of Differentiated Mesenchymal Stem Cells for Treatment of Osteoarthritis

**DOI:** 10.3390/ijms160714961

**Published:** 2015-07-02

**Authors:** Onju Ham, Chang Youn Lee, Ran Kim, Jihyun Lee, Sekyung Oh, Min Young Lee, Jongmin Kim, Ki-Chul Hwang, Lee-So Maeng, Woochul Chang

**Affiliations:** 1 Catholic Kwandong University International St. Mary’s Hospital, Incheon 404-834, Korea; E-Mails: onju1336@gmail.com (O.H.); kchwang@cku.ac.kr (K.-C.H.); 2 Department of Integrated Omics for Biomedical Sciences, Yonsei University, 50 Yonsei-ro, Seodamun-gu, Seoul 120-759, Korea; E-Mail:cylee083@gmail.com; 3 Department of Biology Education, College of Education, Pusan National University, Busan 609-735, Korea; E-Mails: kimran2448@naver.com (R.K.); wlgus3045@nate.com (J.L.); 4 Department of Neurology and Neurological Sciences, Stanford University School of Medicine, Stanford, CA 94305, USA; E-Mail: ohskjhmi@gmail.com; 5 Department of Molecular Physiology, College of Pharmacy, Kyungpook National University, Daegu 702-701, Korea; E-Mail: vetmedic@knu.ac.kr; 6 Department of Life Systems, Sookmyung Women’s University, Seoul 140-742, Korea; E-Mail: jkim@sookmyung.ac.kr; 7 Institute of Catholic Integrative Medicine, Incheon St. Mary’s Hospital, the Catholic University of Korea, College of Medicine, Incheon 403-720, Korea

**Keywords:** osteoarthritis, mesenchymal stem cells, microRNA, small molecule, differentiation

## Abstract

Osteoarthritis (OA) is a chronic, progressive, and irreversible degenerative joint disease. Conventional OA treatments often result in complications such as pain and limited activity. However, transplantation of mesenchymal stem cells (MSCs) has several beneficial effects such as paracrine effects, anti-inflammatory activity, and immunomodulatory capacity. In addition, MSCs can be differentiated into several cell types, including chondrocytes, osteocytes, endothelia, and adipocytes. Thus, transplantation of MSCs is a suggested therapeutic tool for treatment of OA. However, transplanted naïve MSCs can cause problems such as heterogeneous populations including differentiated MSCs and undifferentiated cells. To overcome this problem, new strategies for inducing differentiation of MSCs are needed. One possibility is the application of microRNA (miRNA) and small molecules, which regulate multiple molecular pathways and cellular processes such as differentiation. Here, we provide insight into possible strategies for cartilage regeneration by transplantation of differentiated MSCs to treat OA patients.

## 1. Introduction

Osteoarthritis (OA) is a common form of chronic degenerative joint disease that is slowly induced in the bone, synovium and muscle by several processes including progressive cartilage deterioration, subchondral bone remodeling, loss of joint space, marginal osteophytosis, and loss of joint function [1,2]. OA is caused by several risk factors including age, obesity, mechanical injuries, and joint trauma, and symptoms of the disease include neuropathic pain, depression, and sleep disorder [3]. However, therapeutic tools and unconventional therapeutic methods such as physical surgery for regeneration of damaged osteocytes are lacking [4,5].

Mesenchymal stem cells (MSCs) have recently been applied for treatment of OA in clinical trials because of their regeneration potential and anti-inflammatory effects [6]. In addition, MSCs are easily found in various tissue sources including bone marrow, adipose cells, the spleen, synovial fluid, and the lungs. These cells can differentiate into several cell types including cardiomyocytes, endothelial cells, adipocytes, chondrocytes, and osteocytes [5,6]. MSCs secrete paracrine factors including cytokines, growth factors, and angiogenic factors, which are capable of stimulating migration and cytoprotection [7]. Although MSCs have several beneficial effects, there are three essential factors to consider in using un-differentiated cells: (1) efficiency of direct differentiation of stem cells into specific cell types; (2) survival rate of transplanted cells; and (3) host environments when they were transplanted because not all differentiated pathway have been discovered yet [8,9,10]. In addition, differentiated MSCs have beneficial effects for replacement of damaged tissue because they have characteristics similar to the host tissue. Several studies suggested that therapeutic effects for treatment of OA were showed by transplantation of chondrogenic differentiated MSCs [10,11,12,13,14]. Thus, differentiation of MSCs into specific chondrogenic cells via their modulation for transplant into patients has the potential for treatment of OA.

Differentiation of stem cells is mediated by intrinsic and extrinsic regulators and modification of extracellular niches, which is generally mediated by “cocktails” that are composed of growth factors, signaling molecules, and/or genetic manipulations [8,15]. However, these approaches have limitations: (1) undefined conditions leading to heterogeneous populations of cells; and (2) unexpected risks of virus-mediated genetic modifications [8]. Thus, other approaches for inducing differentiation of stem cells were needed.

Small molecules are useful tools for regulating cell fate and function by inhibiting or activating their specific target signal pathway and mechanism [8]. Moreover, small molecules have several advantages over other techniques, such as gene manipulation, preconditioning, and pretreatment with effectors including growth factors and cytokines: (1) rapid, reversible, and dose-dependent response to biological effects; (2) functional optimization; (3) regulation of specific targets; and (4) temporal control; it regulates specific timing of developmental processes [16,17]. Recently, it has been suggested that a number of small molecules regulate stem cell fate.

The regulation that is activation or suppression of specific gene by transcriptional, post-transcriptional, and translational mechanisms depends on complex networks involving feedback mechanisms, in which microRNAs (miRNAs) are key player [18]. miRNA is small (~22 nucleotides) and non-coding RNA that regulates target gene expression by binding to the 3′ untranslated region (UTR). miRNA plays important roles in regulation of cell proliferation, apoptosis, and differentiation, as well as regulation of MSCs characteristics such as paracrine effects, maintenance, and differentiation [19].

This review was conducted to evaluate the possibility of using differentiated MSCs as a therapeutic tool for treatment of OA, and to discuss the potential to use miRNA or small molecules to induce differentiation of MSCs into chondrocytes.

## 2. Osteoarthritis and Therapeutic Strategies

### 2.1. Osteoarthritis

Chronic disability in people over 50 years of age is strongly associated with disorders of the musculoskeletal system [20]. Osteoarthritis (OA), which is known as a chronic, progressive, and irreversible degenerative joint disease, is by far the most common leading to adult disability [21]. The disease is a complex condition with broad pathology, and is often characterized as a biomechanical disease associated with abnormal joint loading resulting from obesity, joint instability or trauma [22]. OA generally develops progressively over several years, in response to the gradual failure of chondrocytes to repair damaged articular cartilage in synovial joints [20,23]. Joints subjected to OA are unable to withstand normal mechanical stress owing to increased synthesis of tissue-destructive proteinases and apoptosis of chondrocytes, as well as generation of insufficient amounts of extracellular matrix [23].

Articular cartilage plays a major role in cushioning the ends of the bones, allowing for the articulation of opposing joint surfaces [20]. Damage to the cartilage is accompanied by changes to the subchondral bone and synovium [24]. The changes include progressive cartilage loss, subchondral bone remodeling, osteophyte formation, and synovial inflammation [25]. Although OA can occur in any synovial joint, it most commonly affects the knees, hips, and hands [26]. OA affects all genders, ages and races, but is most common in elderly and obese individuals [27]. Symptoms of the disease include joint pain, stiffness and tenderness [27], as well as insomnia, restless leg syndrome, and hypersomnia [28]. In addition, as the cartilage decreases, the bone surface may be affected, resulting in development of osteophytes and direct bone-bone contact [27]. This leads to restricted motion owing to stiffness of the joint, which patients try to avoid by minimizing joint movement; however, this causes muscle atrophy and laxity of the ligaments [29]. But there is currently no cure for OA, and there are no therapies which slow or arrest OA progression [30]. In addition, damaged articular cartilage has limited or no healing capacity with pains and loss of functions [20,31]. Because of these clinical features, treatment of OA requires interventions such as non-cell based and cell based therapies.

### 2.2. Non-Cell-Based Treatments of OA

Although there are no curative therapies currently for OA, some treatments are available to help relieve pain and stiffness, and to maintain functional status [32,33]. Using osteoarthritis medications, which consist of acetaminophen, nonsteroidal anti-inflammatory drugs (NSAIDs), and opioid analgesics, has been suggested as a non-cell-based therapy [34]. Surgery, which is another non-cell based treatment, is reserved as a last resort effort to manage OA symptoms in patients with refractory disease [34]. Surgical techniques consists of arthroscopy, cartilage repair, marrow stimulation by microfracture, abrasion or drilling of the subchondral bone plate, total joint arthroplasty, and osteochondral grafting [33,35,36].

#### 2.2.1. Pharmacologic Treatments

Pharmacologic treatments effectively alleviate persistent symptoms associated with OA [34,37]. Acetaminophen and NSAIDs are effective for mild-to-moderate pain associated with OA [37]. NSAIDs are more effective than acetaminophen for pain relief in OA; however, based on the side effect profile of NSAIDs, acetaminophen is preferred as first-line therapy [34]. Opioid analgesics significantly reduced the intensity of pain [34]. However, these drugs have a potential for serious side effects including acute liver failure, gastrointestinal, renal, and cardiovascular toxicity, and lethargy or nausea/vomiting [34,38]. They also had only moderate effects on physical function. The efficacy of some medications remains controversial. Symptoms and pains can be persistent despite these pharmacological treatments [34].

#### 2.2.2. Surgical Treatments

Arthroscopic techniques include lavage and debridement shaving of rough cartilage to remove debris and inflammatory cytokines [39,40]. However, there is a lack of evidence demonstrating that these methods have significant benefits [40]. Some studies have shown that arthroscopy showed no benefit relative to sham surgery [39,41].

Damaged articular cartilage surface, which has only limited or no healing capacity, relies on cartilage repair techniques [42]. Despite proposed techniques to repair the cartilage surface, such methods can only be applied to focal cartilage defects, which can be seen as a precursor of OA [42].

Marrow stimulating techniques are widely applied to promote chondrogenesis of pluripotent stem cells from subchondral bone marrow in the defect area [39]. The procedures may enhance the expression of secreted factors and cytokines to promote cartilage repair [35]. The techniques enhance chondral resurfacing and take advantage of the healing potential of the body, but limit hyaline repair tissue, variable repair cartilage volume, and possible functional deterioration [39,43].

Arthroplasty is the most widely used orthopedic technique to relieve pain, increase mobility, and improve function in patients by replacing damaged cartilage and bone in the tibiofemoral and patellofemoral joints [44]. The synthetic prosthesis is made of durable, wear-resistant polyethylene, and metal/ceramic is used to balance strength requirements with biocompatibility needs [45]. The main goal of using the bearing inserts, which have some freedom of movement, is to decrease contact stresses at the implant interface [46]. Studies about substitution of damaged joints with artificial prostheses are still very much works in progress, including materials, designs of artificial joints, and surgical techniques. Pain relief in patients after surgery is the most dramatic, rapidly realized result [44]. However, infection, which is potentially the most serious of complications, is a major fault of this total prosthetic replacement [47]. The reported incidence of deep infection around a variety of knee prostheses occurred with a maximum range of 23%, and an average of 5% [47].

The common disadvantages of these surgical techniques include side effects, unsatisfactory progress, high cost for each procedure, and their invasive nature [39]. Although osteochondral transplantation can be autologous or allogeneic for reconstruction of a cartilaginous surface or of osteocartilaginous defects, limited graft availability and technical difficulties hamper this procedure [39].

### 2.3. Non Stem Cell-Based Therapy

Cell-based therapy presents an alternative method for treatment of OA, and may be further subdivided into non-stem cell therapy or stem cell therapy [48]. For first-generation non-stem cell therapy, cultured autologous chondrocyte implantation (ACI) has commonly been applied to treat cartilage defects, and encouraging clinical outcomes have been established [48]. ACI involves chondrocyte isolation from cartilage in non-weight bearing areas, expansion *ex vivo*, and implantation into defective areas in an injectable medium [49]. This first-generation therapy results in significant improvement in function, reduction in symptoms, and the regeneration of cartilage [50,51].

Despite the many positive advantages, first- and second-generation ACI have been limited in technical challenges [51]. In recent years, this technique has been more widely applied as third-generation technique owing to advancements that have improved efficiency rather than injection as a cell suspension [48]. This advanced technique was named matrix-induced ACI (MACI) and involves the attachment or seeding of cultured autologous chondrocytes onto the surface of a biodegradable type I/III collagen membrane or the penetration of cultured autologous chondrocytes within a 3-dimensional scaffold or fleece [52]. Some studies have reported positive outcomes following application of these techniques to knee and ankle lesions [53,54]. The MACI requires less surgical time compared with first- and second-generation ACI, develops less postoperative complications, and can be used to access difficult-to-reach defect sites [52].

However, these non-stem cell therapies require two invasive surgical procedures, and are not cost-effective because they are cell culture-based [55]. Moreover, they have been limited to use for focal cartilage defects, and generalized cartilage loss seen in OA is not an indication for cell implantation [56]. Loss of capacity to generate hyaline cartilage-like extracellular matrix due to chondrocyte de-differentiation and chondrocyte senescence is also a concern [57].

### 2.4. MSC Therapy

#### 2.4.1. MSCs for Cartilage Repair

MSCs are an attractive alternative candidate for regenerative medicine [58]. These cells have the capacity for rapid proliferation and self-renewal, as well as multi-lineage potential that allows differentiation into chondrogenic, osteogenic, and adipogenic pathways [59]. MSCs have recently been suggested as a new cell source for OA treatment in accordance with their ability to differentiate into chondrocytes and the paracrine effects of secreted bioactive substances [60,61]. The anti-inflammatory and immunomodulatory effects of MSCs may also retard the progression of OA [48]. Recent commercial MSC-based therapies for OA in which a suspension of MSCs is injected into the osteoarthritic lesions have been developed [62].

Although considerable and successful results have been reported, many questions still exist, such as which tissue MSCs are suitable or what conditions are appropriate for cartilage repair [35]. The doubtful points limit clinical applications for cartilage injury repair, and as a result, very few clinical studies of direct MSC transplantation have been reported. Alternatively, MSCs can be implanted in a scaffold, encapsulated, or injected in combination with other anti-inflammatory and pro-chondrogenic factors to enhance cell retention and survival [26]. The sources of MSCs can be associated with bone marrow, synovium, adipose tissue and umbilical cords from OA patients or healthy donors [26,27].

#### 2.4.2. Application of SF-MSCs

To explore methods of treating OA, synovial fluid derived-MSCs (SF-MSCs) have been proposed as a source of MSCs involved in putative cartilage repair processes [63]. SF-MSCs, which can be isolated from synovial fluid, are an attractive cell source for OA treatment because of their high proliferative activity and chondrogenic potential relative to other tissue derived-MSCs [64]. SF-MSCs existed more in OA than in other arthropathies, suggesting their possible role in the pathophysiology of arthritis [65]. The cells from OA patients are already specific for the patients’ bodies [26], and they can be easily harvested from OA patients during arthrocentesis or routine arthroscopic examination without damaging normal tissues [66]. For repair processes, the SF microenvironment of OA patients is able to enhance their proliferative potential, boosting MSCs proliferative response even further [63]. MSCs from synovium expanded much faster than those from bone marrow when cultured with autologous human serum. Previous studies have already suggested the potential for SF-MSCs to mitigate OA. The direct articular injection of MSCs from synovium promoted cartilage regeneration with low invasion, without periosteal coverage and a scaffold in rabbit [67]. Moreover, transplantation of synovium MSCs contributed to meniscal healing in micominipigs [64]. Finally, synovial tissue may serve as a reservoir of MSCs that migrate to the site to participate in the repair response following intra-articular tissue diseases [68].

## 3. Differentiation of MSCs into Chondrogenic Cells

One of the best strategies for the treatment of OA is ACI [48]. However, ACI requires an invasive surgical procedure which have limitations in ensuring the required number of cells. Moreover, patient-derived chondrocytes affect the treatment according to the age and health of the patient [69]. Treatment using stem cells to solve these problems is needed. However, transplantation of non-differentiated MSCs is associated with problems such as heterogenic populations of cells [7,8,9]. One of the strategies to resolve these problems is use of chondrogenic differentiated MSC.

### 3.1. Regulation of Differentiation of Stem Cells by miRNA

According to a recent report, miRNAs play important roles in various biological phenomena, and in particular, miRNAs play a critical regulator of chondrogenic differentiation of stem cells [70]. miRNA regulates the intracellular signaling pathway by inhibiting the expression of specific target genes [71], resulting in regulation of a variety of biological changes. During differentiation of human MSCs into chondrocytes, some types of miRNA show abnormal expression patterns. For example, miR-23b [45], miR-140 [72,73], miR-455 [73], and miR-335 [74] positively regulate chondrogenic differentiation, while miR-29a [75], miR-193b [76], and miR-574 [77] negatively regulate chondrogenesis.

#### 3.1.1. Stimulation of Differentiation of Stem Cells into Chondrocytes

Miyaki *et al.* [72] reported that miR-140 expression increased in chondrogenic differentiated human BM-MSCs. Moreover, they demonstrated abnormally reduced miR-140 expression in OA cartilage and an apparent correlation with increased ADAMTS5 expression and reduced COL2A1 expression [72]. Ham *et al.* reported that miRNA-23b induced differentiation of human bone marrow-derived-MSCs [45] and synovial fluid-derived-MSCs [66] into chondrocytes via regulation of protein kinase A signaling. miR-23b increased expression of the chondrocyte markers of collagen type II, collagen type X, and Sox9, while it reduced hypertphic marker of MMP-2/-9.

#### 3.1.2. Inhibition of Differentiation of Stem Cells into Chondrocytes

In addition, Guérit *et al.* [77] reported that miR-574 inhibited chondrogenesis by targeting RXRα. Specifically, they revealed the importance of miR-574 to MSCs maintenance [77]. They also found that miR-29a repression by Sox9 stabilized Foxo3a, while HDAC4 promoted chondrocyte formation [77]. Similarly, miR-193b inhibited early chondrogenesis by targeting TGFB2 and TGFBR3, as well as regulating inflammation through repression of TNF-α [76]. Thus, based on the number of reported miRNA, it has proven an important factor in chondrogenic differentiation (Table 1).

**Table 1:** Summary of chondrogenic differentiation by miRNA.

Cell Type	miRNA	Target Gene	Function	References
human MSCs	miR-23b	*PRKACB*	Positive regulation of chondrogenesis	[45]
human MSCs	miR-140	*ADAMTS5*	Positive regulation of chondrogenesis	[72,73]
ATDC5	miR-455	*Smad2/3*	Positive regulation of chondrogenesis	[73]
Mouse MSCs	miR-335	*Daam1*, *ROCK1*	Positive regulation of chondrogenesis	[74]
human MSCs	miR-29a	*FOXO9*	Negative regulation of chondrogenesis	[75]
human MSCs	miR-193b	*TGFB2*, *TGFBR3*	Negative regulation of chondrogenesis	[76]
human MSCs	miR-574	*RXRα*	Negative regulation of chondrogenesis	[77]

#### 3.1.3. Limitations of Using miRNAs

Although miRNA regulates several genes, miRNA still has problems to be solved. First, miRNAs can target hundreds of genes [78,79]. This can be an advantage and a disadvantage. This feature has a synergistic effect by controlling the various targets involved in the physiological and pathological changes. Conversely, this feature is very complex in terms of understanding the mechanism of action compared to the cytokine or small molecule compound previously reported. Second, it is difficult to deliver *in vivo*. This corresponds both to the RNA interference-based therapeutics, as well as miRNA. miRNA is a promising treatment for OA, but its disadvantages must be overcome through further study.

### 3.2. Induction of Differentiation by Small Molecules

Small molecules are critical to elucidation of the mechanism and development processes through which inhibition or activation of target molecules occurs. Such molecules influence DNA replication, differentiation, migration, and apoptosis by controlling the intracellular signaling pathways. Small molecules are also important to the induction of stem cell differentiation in a variety of cell types including cardiomyocytes [80], adipocytes [81], and hepatocytes [82], and they are known to be involved in cell reprogramming.

In our previous study, we found that treatment with H-89, a protein kinase A (PKA) inhibitor, promoted chondrogenic differentiation of human bone-marrow-derived MSCs. In addition to the increase in miR-23b induced by H-89, it promoted chondrogenesis through targeting of PRKACB [44]. Hara *et al.* [83] reported that harmine induced chondrogenic differentiation through increases in CCN2, SOX-9, aggrecan, and COL2α1 levels. Therefore, harmine can be a useful compound for prevention and/or regeneration of cartilage degradation in response to aging or OA [83]. Eslaminejad *et al.* [84] reported that BIO at 0.01 µM could accelerate chondrocyte differentiation. The study revealed that the expression of cartilage-specific genes including Sox9, aggrecan, and collagen II was increased in BIO-treated cells at day 14, whereas the expression level of these genes reached a maximum at day 21 in non-treated cell [84]. Cho *et al.* [85] studied the fact that 5{i,2} induced chondrogenesis in hMSCs which is based on a Δ^5^-2-oxopiperazine structure. In addition, faster chondrogenic differentiation was detected in 5{i,2}-treated in MSCs compared to TGF-treated cells [85]. Henderson *et al.* [86] found that *all-trans* retinoic acid influenced differentiation of MSCs into chondrocytes by regulating Smad and p38 signal pathway [86]. Moreover, Pevsner-Fischer *et al.* [87] suggested that Pam3cys, a prototypic Toll-like receptor (TLR)-2 ligand, induced nuclear factor-κB (NF-κB) translocation, secreted interleukin (IL)-6, decreased MSC motility, and up-regulated MSC proliferation. The author suggested that TLR ligands have the capacity to inhibit differentiation of MSCs into mesodermal cell lines [87] (Table 2).

**Table 2:** Summary of chondrogenic differentiation by small molecules.

Cell Type	Small Molecule	Target Gene	Function	References
human MSCs	H-89	PKA inhibitor	Positive regulation of chondrogenesis	[45]
ATDC5	harmine	Inducer of CCN2	Positive regulation of chondrogenesis	[83]
mouse MSCs	BIO	Activation of Wnt signal pathway	Positive regulation of chondrogenesis	[84]
human MSCs	5{i,2}	Δ^5^-2-oxopiperazine core structure	Positive regulation of chondrogenesis	[85]
MSCs	*all-trans* retinoic acid	Smad/p38	Positive regulation of chondrogenesis	[86]
mouse MSCs	Pam3cys	NF-κB	Negative regulation of chondrogenesis	[87]

### 3.3. Others

There are many factors regulating differentiation of stem cells into chondrocyte, including cytokine, hormone, growth factor, and so on. They have variant effect to regulate biological processes, not only differentiation but also proliferation, apoptosis, and maintenance of cells.

#### Induction of Differentiation of Stem Cells by Cytokines or Growth Factors

Cytokines are important factors for regulating various biological processes. Jagielski *et al.* [88]. showed that integrin-10 (IL-10), or Tumor Necrosis Factor (TNF) α, induced differentiation of MSCs in 3D high-density (H-D) culture into chondrocytes [88].They detected expression of chondrogenic genes, including type II collagenase, sox9, aggrecan, and TNFα, that was increased in IL-10- or TNFα-treated MSCs [88]. Huang *et al.* [89] demonstrated that tumor growth factor (TGF) β stimulated chondrogenic differentiation by regulating histon deactylase (HDAC) 1 [89]. The author suggested that HDAC1 induced chondrogenic differentiation by inhibiting canonical Wnt/β-catenine signal pathway. Zhang *et al.* [90] also suggested that TGFβ induced chondrogenic differentiation. The author suggested that TGFβ/SMAD pathway and IL-1β were involved in differentiation of stem cells indo chondrocyte and hypertrophy. They suggested that deferral dynamic compression (activation of TGFβ/Activin/Nodal signal pathway, and suppression of BMP signaling) induced cartilage formation and suppressed chondrocyte hypertrophy [90]. All growth factors have no effects on differentiating chondrocytes. Prostaglandin F2α receptor (FP) signaling also promote chondrogenic differentiation through bone morphogenic factor (BMP) signaling [91]. Huang *et al.* found that nerve growth factor (NGF) decelerates chodrogenic differentiation by binding its high affinity receptor, tropomyosin receptor kinase A (TrkA), in ATDC5 cells [92].

## 4. Strategies for Transplantation of Stem Cells into the OA Area

Tissue engineering with chondrocytes and MSCs is now considered to be a promising way of repairing articular cartilage lesions. For treatment of OA, several clinical trials tried direct transplantation of MSC, using a scaffold with MSCs, only MSCs, or MSCs mixed with cytokines and/or growth factors, into knee OA.

### 4.1. Transplantation of MSCs with Scaffolds

Scaffolds were used to improve cell attachment, growth, nutrition supply in microenvironment of the transplanted cells, in varieties including collagen, fibrin and hyaluronic acid. It was used to have the holding substrate and the microenvironment of the cell carrier form a gel or a 3D structure. Kayakabe *et al.* [93] reported that autologous transplantation of rabbit BMMSC with a hyaluronic acid gel sponge can effectively regenerate osteochondral defects in cell therapy [93]. After 12 weeks of MSC transplantation with a hyaluronic acid gel sponge, well-repaired cartilage resembling articular cartilage in the surrounding structure was observed. As a result, they reported that the hyaluronic acid gel sponge influences chondrogenic differentiation. Wakitani *et al.* [94] also reported on the use of transplanted MSCs seeded within collagen type I hydrogels to repair isolated, full-thickness, cartilage defects in humans [94]. Guo *et al.* [95] tried to treat for OA through transplantation of autologous MSCs into bioceramic scaffold-β-tricalcium phosphate (β-TCP). They found that the method was an effective strategy for treatment of OA [95].

### 4.2. Direct Injection of MSCs

Local delivery of MSCs has beneficial effects including enhancement of joint repair and reduction of the degenerative changes related to OA and the method is the simplest approach for treatment of OA [21,96]. Gao *et al.* [97] showed the therapeutic effects of fibroblast growth factor receptor (FGFR) 3^+/+^ MSCs which were transplanted into the OA area intra-femorally in FGFR 3^−/−^ mice. They suggested that transplanted cells were differentiated into osteocytes, but they were concerned that transplanted cells could migrate to other areas [97]. Murphy *et al.* also detected the therapeutic effects of transplanted human BMSCs in a caprine model of OA. They suggested that cells implanted by local delivery stimulated regeneration of meniscal tissue and reduced progressive destruction in injured area [98]. Centeno *et al.* [99] studied the fact that implantation of autologous MSCs have the capacity to stimulate cartilage growth and to decrease pain in degenerative joint disease. The effects of transplanted MSCs were detected by MRI for 24 weeks [99].

### 4.3. Mixed Injection (Stem Cells Combined with Growth Factors, Cytokines, or Scaffold)

There were several strategies to improve the therapeutic effects by transplantation of MSCs by mixing cytokines or growth factors with scaffolds. Mrugala *et al.* [100] tried transplantation of ovine MSCs (oMSCs) with or without chitosan and with or without TGFβ3 in a fibrin clot. They demonstrated that transplantation of oMSCs mixed with chitosan and TGFβ3 had effects in terms of treatment for OA [100]. Platelet-rich plasma (PRP) is an autologous enriched source of chondrogenic growth factors including TGF-β, and platelet-derived growth factor (PDGF) so that PRP can be used as a therapeutic source for treatment of osteochondral defection [101]. MSC transplantation on platelet-rich fibrin glue led to the RHSSK score of the patients improving, and the MRI results revealed complete defect fill and complete surface congruity with native cartilage. Seo *et al.* [101] demonstrated the therapeutic effects of bilayer gelatin/β-TCP (GT) combined with stem cells, chondrocyte, BMP-2, and PRP (Ch/MSC/PRP/GT) on osteochondral defects of the talus in horse. They confirmed that Ch/MSC/PRP/GT stimulated osteochondral regeneration and had a capacity as a useful source for treatment of OA [101]. Haleem *et al.* [102] suggested that autologous BMMSC transplantation on platelet-rich fibrin glue as a cell scaffold may be an effective approach to promote the repair of articular cartilage defects [102].

## 5. Conclusions

This review suggests methods for treatment of OA, namely, transplantation of stem cells and differentiated MSCs using miRNA, small molecules, growth factors, and cytokines. miRNA and small molecules have regulatory effects on transcription factors and can target specific molecules, enabling them to be used to induce differentiation of stem cells into chondrogenic cells. Although various studies have suggested methods of differentiation, there are many unknown factors that may be able to induce differentiation of stem cells into chondrocytes. Thus, additional studies should be conducted to identify new factors capable of differentiating stem cells into chondrogenic cells. Moreover, implantation strategies which were stem cells combined with or without scaffolds, growth factor, or cytokine need improved therapeutic effects for treatment of OA.
